# Expedition into Taurine Biology: Structural Insights and Therapeutic Perspective of Taurine in Neurodegenerative Diseases

**DOI:** 10.3390/biom10060863

**Published:** 2020-06-05

**Authors:** Mujtaba Aamir Bhat, Khurshid Ahmad, Mohd Sajjad Ahmad Khan, Mudasir Ahmad Bhat, Ahmad Almatroudi, Safikur Rahman, Arif Tasleem Jan

**Affiliations:** 1 School of Biosciences and Biotechnology, Baba Ghulam Shah Badshah University, Rajouri 185234, India; mujtaba868@gmail.com (M.A.B.); bhatmudasirrashid@gmail.com (M.A.B.); 2 Department of Medical Biotechnology, Yeungnam University, Gyeongsan 38541, Korea; ahmadkhursheed2008@gmail.com; 3 Department of Basic Sciences, Imam Abdulrahman Bin Faisal University, Dammam 31451, Saudi Arabia; khanmsa@hotmail.com; 4 Department of Medical Laboratories, College of Applied Medical Sciences, Qassim University, Qassim 51431, Saudi Arabia; aamtrody@qu.edu.sa; 5 Munshi Singh College, BR Ambedkar Bihar University, Muzaffarpur, Bihar 845401, India

**Keywords:** aggregation, neurodegenerative diseases, osmolytes, protein folding, therapeutics, unfolded protein response

## Abstract

Neurodegenerative diseases (NDs) are characterized by the accumulation of misfolded proteins. The hallmarks of protein aggregation in NDs proceed with impairment in the mitochondrial function, besides causing an enhancement in endoplasmic reticulum (ER) stress, neuroinflammation and synaptic loss. As accumulation of misfolded proteins hampers normal neuronal functions, it triggers ER stress, which leads to the activation of downstream effectors formulating events along the signaling cascade—referred to as unfolded protein response (UPRER) —thereby controlling cellular gene expression. The absence of disease-modifying therapeutic targets in different NDs, and the exponential increase in the number of cases, makes it critical to explore new approaches to treating these devastating diseases. In one such approach, osmolytes (low molecular weight substances), such as taurine have been found to promote protein folding under stress conditions, thereby averting aggregation of the misfolded proteins. Maintaining the structural integrity of the protein, taurine-mediated resumption of protein folding prompts a shift in folding homeostasis more towards functionality than towards aggregation and degradation. Together, taurine enacts protection in NDs by causing misfolded proteins to refold, so as to regain their stability and functionality. The present study provides recent and useful insights into understanding the progression of NDs, besides summarizing the genetics of NDs in correlation with mitochondrial dysfunction, ER stress, neuroinflammation and synaptic loss. It also highlights the structural and functional aspects of taurine in imparting protection against the aggregation/misfolding of proteins, thereby shifting the focus more towards the development of effective therapeutic modules that could avert the development of NDs.

## 1. Introduction

The human brain is a complex organ of the human body, consisting of different cells, such as neurons, oligodendrocytes, microglia, astrocytes, etc., that work in a coordinated manner and regulate spatiotemporally. The underlying homeostasis network that regulates the complex architectural of the brain, being robust at the beginning (young age), shows a gradual decline in terms of functioning with age, thereby resulting in cognitive decline and, as such, development of a variety of neurodegenerative diseases (NDs) [1]. The lack, on the therapeutic front, of treatments for slowing the rate of occurrence of NDs, these often become devastating, not only for the patients and their families (for care and dependency), but often also leaving a deep scar in terms of the mounting economic burden. With huge socioeconomic constraints, the etiology of NDs [Alzheimer’s disease (AD), Parkinson’s disease (PD), Amyotrophic lateral sclerosis (ALS) and others] has become a burning issue that needs properly addressing, not only in terms of understanding the disease mechanism, but also in terms of the advancement of developing potential treatment regimes that can control the progression of the NDs [2,3,4,5,6,7].

In view of the hallmarks of NDs, taurine displays a series of beneficial effects that appear promising in combating the menace of NDs. Of these, taurine stabilizes membrane proteins(and as such maintains their structural integrity [8,9,10]), reduces apoptosis by modulating neuroinflammatory responses [11,12], exerts antioxidant effects by reducing ischemic and traumatic insults [13,14], exerts neuromodulatory effects by acting as an agonist of GABA and glycine receptors [15], and improves different correlates of memory [16,17], thereby augmenting learning and memory process [18,19,20]. The absence of disease-modifying therapeutic targets in different NDs, and the exponential increase in the number of cases, makes it critical to explore new approaches to treating these devastating NDs. The present article contains recent and useful information pertaining to the etiology of NDs and factors that contribute to the development of NDs (in particular mitochondrial dysfunction, endoplasmic reticulum stress, neuroinflammation and synaptic loss, which progress to neuronal death), together with the biology of taurine, a major cellular osmoprotectant that overcomes the toxicity that arises due to aggregation of misfolded proteins. Taurine thereby imparts protection against oxidative stress, via regulation of protein folding/unfolding.

## 2. Neurodegeneration

The pattern of vulnerability that turns resilient neurons into susceptible ones differs among NDs. AD is a progressive form of ND that shows either familial (FAD; early onset) or sporadic (SAD; showing late onset) origin [3]. FAD accounts for 5% of AD cases, and arises predominantly due to mutations in the amyloid precursor protein (APP) and Presenilin 1 and 2 (PS1 and PS2) [21,22,23,24,25,26]. Mutations lead to the formation and thus accumulation of neurofibrillary tangles (tau tangles; hyperphosphorylated tau protein) and plaques of amyloid-β (Aβ) in and around neurons, causing synaptic impairment and neuronal death, which ultimately leads to cognitive alteration and behavioral changes [2,3,7,27,28,29,30,31,32,33]. However, late onset of AD (LOAD), accounting for 90% of Alzheimer’s cases, is believed to have many risk factors associated particularly with ageing, genetic polymorphism at different gene loci (such as R47H mutation in Trem2), and the presence of ApoE4 allele [34,35]. PD has well-established, environmentally acquired and genetic risk factors associated with it. PD shows an early onset that arises due to mutations in Parkin (PARK2)- and PTEN-induced putative kinase (PINK1), while late onset PD arises due to mutations in α-synuclein (SCNA), ubiquitin C-terminal hydrolase L1 and leucine rich repeat kinase 2 (LRRK2) genes, leading to the formation of Lewy bodies (LBs; accumulation of α-synuclein and parkin substrate) [36,37,38,39,40]. Prion disease is caused by the misfolding of the prion proteins. With major contributions from sporadic types, 5–15% of cases show a genetic predisposition, with mutations in the prion protein gene (PRNP) located on chromosome 20 in humans [40]. Prions, being infective entities, are capable of triggering the refolding, and thus aggregation, of native proteins to oligomers and fibrils [41]. Misfolding of Cu/Zn superoxide dismutase (Cu/Zn SOD) is a characteristic feature of both familial and sporadic form of ALS [42,43,44]. A highly conserved nuclear protein TDP43 encoded by the TARDBP gene also contributes significantly to the occurrence of ALS [3,45].

**Figure 1:**
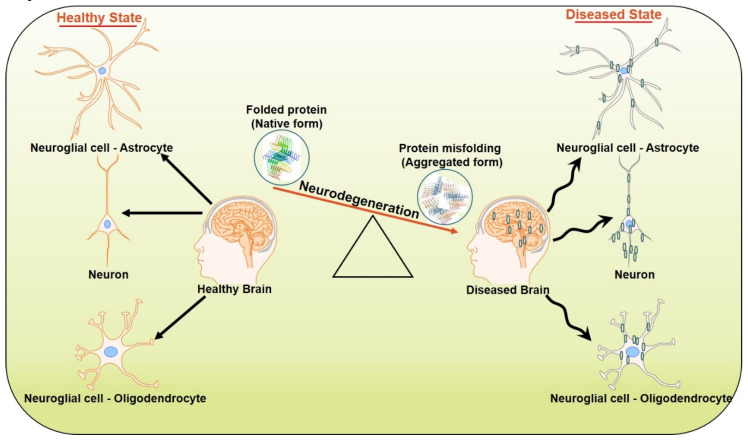
Progression of neurodegenerative diseases.

As a major hallmark of NDs, protein aggregation, and their accumulation in different parts of the central nervous system (CNS), hinders not only the synaptic transmission process, but also impairs mitochondrial function, along with enhancing endoplasmic reticulum (ER) stress [46,47] (Figure 1). The information on NDs in association with mitochondrial dysfunction, ER stress, neuroinflammation and synaptic loss that ultimately lead to neuronal death, are discussed with respect to their involvement in AD. 

### 2.1. Mitochondrial Dysfunction

As a vital cellular organ, mitochondria are associated with the regulation of cellular metabolism. Performing vital functions, impairment of the enzymatic machinery, particularly of the tricarboxylic acid cycle, hampers the functions of the mitochondria processes and also affects the overall functionality of the cell. Acting as a source of energy, impairment of the mitochondrial enzymatic machinery has the consequences of reducing energy metabolism in the brain. Studies of mitochondrial functioning in the AD brain revealed a greater extent of impairment in the functioning of the pyruvate dehydrogenase and α-ketoglutarate dehydrogenase complexes, followed by isocitrate dehydrogenase [48,49]. Impairment in the mitochondrial enzymes, which leads to an imbalance in the energy status of brain, often has serious consequences for brain functioning (damage of neurons), and thereby for the development of neurodegeneration [50,51,52]. 

**Figure 2:**
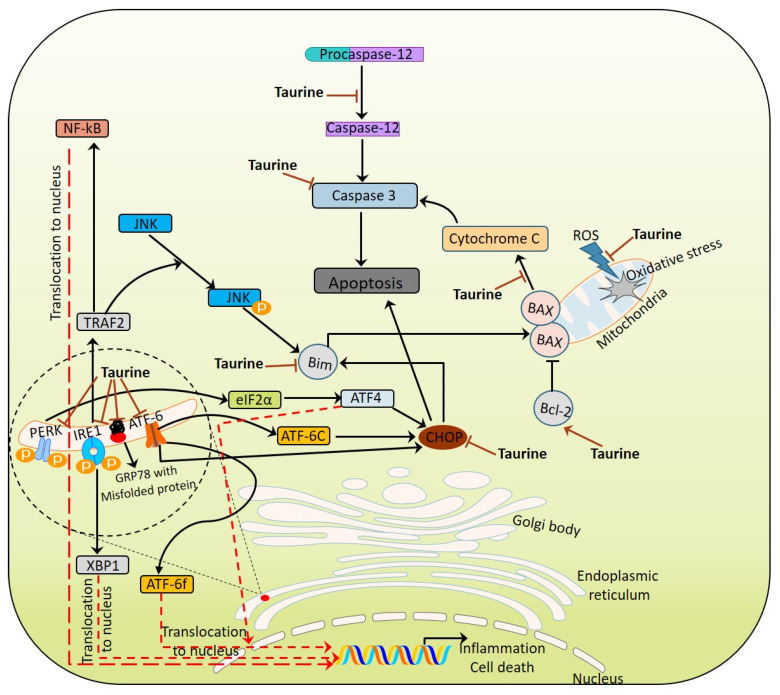
Taurine combatting Mitochondria—Endoplasmic Reticulum stress module.

Increase in Aβ aggregation and deposition leads to oxidative damage via the enhancement of the production of H_2_O_2_ [53]. Aβ accumulation in the synaptic mitochondria leads to high levels of cyclophilin D [CypD; mitochondrial permeability transition pore (mPTP)], which causes significant changes in synaptic Ca^2+^ [54]. Translocation of CypD from the matrix to mPTP (CypD–mPTP) increases its interaction with adenine nucleotide translocase, resulting in the collapse of membrane potential via the opening of the pore, and thereby leads to the death of the neurons. Additionally, inhibition of the mitochondrial electron transport chain triggers the production of ROS (Reactive Oxygen Species), capable of damaging proteins, lipids and nucleic acids. Increased production of ROS acts as a trigger for autophagy, which subjects mitochondria to mitophagy [54,55] (Figure 2).

### 2.2. ER Stress

As a vital cellular organ, ER is involved in the synthesis of proteins. Any disruption in the ER synthetic machinery causes unfolded or misfolded proteins to accumulate in the lumen of the ER [56,57]. The cell has a unique way of trafficking unfolded or misfolded proteins to the cytoplasm for degradation, in a process referred to as ER-associated protein degradation (ERAD) [58,59]. Saturation in the unfolded or misfolded protein trafficking machinery leads to ER stress, which elicits a dynamic signaling cascade referred to as unfolded protein response (UPR^ER^) [4,5,60,61,62,63]. UPR^ER^ is mediated by three transmembrane proteins, inositol requiring enzymes-1 (IRE-1), PKR like ER kinase (PERK) and activating transcription factor-6 (ATF6),which act as stress sensors (triggers signaling downstream via transcription factors) in the ER [56,63,64]. In the normal state, the luminal domains of stress sensors remain bound with a chaperone, BiP (Binding immunoglobulin protein), thereby inhibiting activity at the surface of the cytosolic domain. During stress, BiP release brings about dimerization of IRE-1 and PERK, that together initiates signaling across the UPR signaling cascade [4,5,61,62]. In the UPR signaling cascade, PERK exerts an inhibitory effect on the eukaryotic translational initiation factor 2α (eIF2α), causing rapid attenuation of the translational event [65]. At the same time, it favors the translation of ATF4 (Activating Transcription Factor-4), capable of controlling the expression of genes related to amino acid metabolism, autophagy and apoptosis [64,66,67,68]. Activation of IRE-1 halts the expression of genes associated with ER protein translocation, lipid synthesis and the folding of proteins, via the splicing of X-box binding protein-1 (XBP-1) [69]. The third transducer of UPR^ER^, ATF6, upon activation is translocated into the Golgi complex, and cleaved by site 1 protease and site 2 protease to release the active N terminus part, which is in turn involved in the upregulation of genes associated with normal ER functioning, such as XBP1, CHOP, etc. [64,66]. In the *Drosophila melanogaster,* AD model, the reduction of Ca^2+^ release from ER stores via *Xbp-1* over-expression imparts protection against Aβ toxicity [70,71]. Together, initial UPR seems protective as it favors the expression of chaperons promoting refolding (degradation in the event of failing to bring about refolding), while prolonged stress conditions trigger additional pathways that in turn lead to cellular apoptosis [72]. 

### 2.3. Neuroinflamation.

Being multifaceted processes, NDs involve different cell types in the brain. Of them, microglia—implicated in the innate immunity of the brain—plays an important role in the progression of NDs, in particular AD [73,74]. Exhibiting a high expression of AD risk factor genes, microglia-mediated increases in proinflammatory cytokines have been reported both from patients with AD and from disease models of the disease, and has been found to contribute to neuronal cell death [75,76]. Activating NLRP3 inflammasome, the aggregation of Aβ and α-syn (α-Synuclein) led to enhanced production of proinflammatory cytokines interleukin (IL)-1β and IL-18 [77,78], the binding to neuronal receptors of which initiates a series of cytotoxic events, i.e., the aberrant influx of calcium and the activation of the JNK (c-Jun N-terminal kinase) signaling pathway [79,80]. Simultaneously, activation of the microglial NLRP3 inflammasome enhances Aβ aggregation and its spread, thereby creating a feedback loop that exacerbates neuronal cell death [81]. Additionally, TNFα production by microglia potentiates neuronal excitotoxicity, which progresses to neuronal cell death via signaling through the death receptors expressed on neurons [82,83].

### 2.4. Synaptic Loss

Referring to the conjunction between the axon of one neuron and the dendritic spine of another neuron, synaptic plasticity (formation and elimination)in neuronal circuits maintains the structure-based long-term potentiation (LTP) essential in memory formation [84,85]. Of the different cell subsets, microglia (constituting 10–15% of brain cells) and astrocytes [major glial cells in the central nervous system (CNS)] provide trophic support to neurons, besides performing roles in the refinement and coordination (synaptogenesis; neurotransmitter release and synaptic transmission)of neural circuits [86,87,88]. In NDs, an accumulation of toxic protein aggregates at synapses causes synaptic dysfunction that often increases the vulnerability of neurons to becoming primed for removal [89,90,91]. Contributing to neural network formation, for shaping brain connectivity, glial subset cell populations (astrocytes and microglia) perform the pruning of weaker synapses in early development ([92,93,94,95] and references therein). Though several pathways—such as the fractalkine pathway, complement pathway, etc.—have been implicated in the synaptic elimination process [94,96,97], the pathological consequences of NDs are observed in response to internal glial defects (genetic mutations) or dysfunctional regulation in the execution of the pathways. It is now well established that astrocytes and microglia play important roles in refining synaptic connections (synaptic elimination) in the context of the development of different NDs. A major hypothetical mechanism involved is the activation of the complement system, preferably C3 and C1q, followed by their active deposition at synaptic terminals, thereby priming aberrant removal (synaptic elimination) [98,99,100,101]. In AD, the accumulation of Aβ at synapses (excitatory) occurs even before its accumulation as plaques in the extracellular milieu, as reported in both mouse and human studies [102,103,104]. The accumulation of oligomeric Aβ at synapses impairs LTP, which progresses with the weakening of the synapse and the induction of synaptotoxicity [105,106]. The effect of synaptic LTP impairment and synaptotoxicity were prevented on administration of C1q neutralizing antibodies, and even in the C1q knockout mouse model [100,107]. Additionally, microglia that lies close to Aβ plaques, upregulating ApoE4 expression in the TREM2 dependent pathway, is found to be associated with enhancement of the synaptic loss [108,109]. Microglia-mediated release of C1q, together with proinflammatory cytokines (TNFα and IL-1α), regulate astrocytic function [110]. Conversely, the astrocyte-mediated release of NF-kB induces Wnt-dependent microglial proliferation; thereby regulating microglial phenotypes [111,112]. Acting together in the efficient remodeling of the synapse, microglia and astrocytes together coordinate in the ensuing efficient remodeling of the synapse.

## 3. Taurine—A Savior

Taurine (2-amino-ethanesulfonic acid) is among the most abundant amino acids in mammals [113,114,115]. The history of taurine dates back to 1827, with its isolation from the bile of Bos Taurus [116]. However, its origin seems more ancient in terms of phylogeny; it is present in higher amounts in algae, absent among viruses and bacteria (except *Bacillus subtilis*, where it serves as a source of carbon, nitrogen and sulfur), found in trace amounts among fungi and plants, and found at higher concentrations among animals [113,117]. Taurine is considered a conditionally essential nutrient for humans [118]. It is considered safe for humans as it does not exert any genotoxic, teratogenic or carcinogenic effect within the human body [13,119,120]. The European Food Safety Authority (EFSA) has set 1000 mg/kg/per day as the No Observed Adverse Effect Level (NOAEL) regarding the consumption of taurine as part of energy drinks [13]. 

Required in large amounts, its requirement among humans is fulfilled by endogenous synthesis, preferentially in the liver and kidneys, or through its procurement as part of the diet [121,122]. As endogenous taurine synthesis does not fulfill the physiological requirements of humans, they rely on dietary supplementation to fulfill their need for taurine. Though colostrum containing high levels of taurine fulfils initial taurine requirement among new-borns, this is followed by supplementation as an addition to infant formulas [121]. Adults fulfill their requirement for taurine via retention in greater amount across different tissues. Categorized as a non-essential amino acid (due to endogenous synthesis), its incorporation into proteins has not been reported. The popularity of taurine comes from its involvement in diverse physiological functions; as a neurotransmitter [123,124], as an osmolyte [125,126,127], as a trophic factor in CNS development [128] and as a neuroprotector in glutamate (Glu)-induced neurotoxicity [129,130], maintaining structural integrity of the membrane [8,9] and regulating calcium homeostasis [131,132]. Additionally, taurine has been found to be involved in modulating inflammation [133,134], and acting as an antioxidant in scavenging free radicals [135,136] and in reducing apoptosis [137,138]. 

## 4. Structure and Physiochemical Properties

Taurine (NH_3_^+^–CH_2_–CH_2_–SO_3_^–^) is a sulfur β-amino acid that resembles, in its structure, an inhibitory neurotransmitter γ-aminobutyric acid (GABA) [122]. In its structure, the amino group (NH_3_^+^) located on the β-carbon and carboxylic (CO_3_^–^) group of amino acids is replaced by a sulfonic (SO_3_^–^) acid group. The presence of sulfonic (SO_3_^–^) acid group attributes taurine with unique physicochemical properties; a pKa value of ~2 (very low; more acidic than aspartate and GABA) for the sulfonic acid group, and a pKb value of 9 for the amine group, which results in the zwitterion state of the molecule at physiological pH [139]. Taurine concentration is higher in plasma (80 µM) and varies greatly among tissues [140]. It undergoes cyclization via the intramolecular hydrogen bond. The cyclic conformation of taurine hinders its transport by passive diffusion across the biological membrane. Transport of taurine across the intestinal surface occurs by either high-affinity Na^+^/Cl^−^ taurine transporter, Tau-T encoded by SLC_6_A_6_ gene, and/or proton (H^+^)-coupled amino acid transporter (PAT1; low affinity but a major taurine transporter) [141,142]. Anderson et al. (2009) reported that PAT1 is a major transporter of taurine during meals, while its counterpart acts as a major transporter at low concentrations, i.e., in between meals [143]. 

## 5. Taurine Biosynthesis 

**Figure 3:**
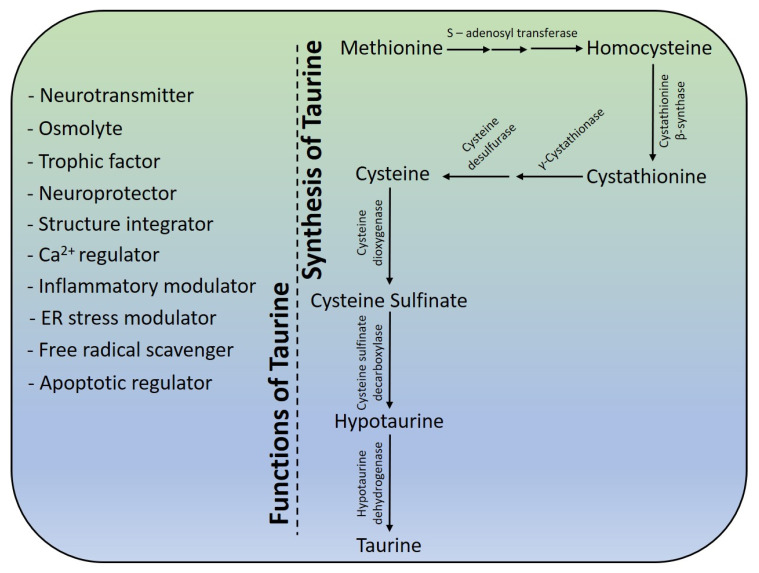
Structural and functional aspects of osmolyte taurine.

The synthesis of taurine occurs from primary metabolites, methionine and cysteine, generated in different metabolic pathways. Synthesis begins with the conversion of methionine to cysteine via S-adenosylmethionine, S-adenosylhomocysteine, homocysteine and cystathionineintermediates [144] (Figure 3).

The formation of cystathionine from homocysteine occurs through a condensation reaction, catalyzed by cystathionine β-synthase in the presence of the serine molecule. The proceeding reactions from cystathionine to cysteine (generated in the pathway or obtained through the diet), and finally to taurine, occurs via cysteine sulfinate and hypotaurine. Low concentrations of enzymes, cysteine dioxygenase (CDO) and cysteine sulfinate decarboxylase (CSAD), catalyzing the conversion of cysteine to hypotaurine via cysteine sulfinate, being rate-limiting, contribute low levels of taurine produced by the endogenous pathway. In the taurine synthesis pathway, vitamin B6 (pyridoxal phosphate) acts as co-factor for three enzymes: cystathionine β-synthase, γ-cystathionase and CSAD [145]. The addition of two minor modifications in the major taurine biosynthesis pathway have also been reported: (1) One that operates in the brain and liver with the diversion of cysteine sulfinic acid to cysteic acid (catalyzed by cysteine sulfonic acid dehydrogenase), and finally to taurine by CSAD; (2) A second that operates in the kidney with the diversion of cysteine to cysteamine via the pantothionate pathway, and finally to hypotaurine by cysteamine dioxygenase [146,147]. 

## 6. Neuroprotective Effects of Taurine

A detailed description of the neuro-developmental effects of taurine are discussed under the following sub-headings and Table 1:

### 6.1. As Antioxidant Molecule

Oxidative stress, which arises from the over-production of ROS, such as hydrogen peroxide (H_2_O_2_), hydroxyl radical (**^·^**OH), superoxide ion (O_2_**^−^**), etc., is known to play an important role in the development of NDs [157,158]. Despite the fact that the cellular protection mechanism offered by taurine is elusive, it is considered as a cellular antioxidant. Its antioxidant property in neutralizing ROS is believed to be attributed to its sulfonic group, as revealed in an in vitro study where it was found to be neutralizing the effect of H_2_O_2_ [159]. It was also found to be attributing protective effect against hypochlorous acid and nitric oxide [136,160,161]. Its indirect protection mechanisms include counter-protection, in reducing the deleterious effect of ROS via alteration of membrane lipid content, which reduces the fluidity of the membrane and as such the efflux of water and ions from the cell [160,162,163]. Additionally, taurine offers protection to cells on exposure to toxins via maintenance of the levels of antioxidant enzymes, such as superoxide dismutase, glutathione peroxidase and thioredoxin reductase [164,165,166,167].

### 6.2. As Stabilizer in Regulating Protein Folding/Unfolding 

The folding of proteins is essential for ensuring the functional state of a protein. Having less influence on the sequence of amino acids, the folding of a polypeptide chain is largely determined by the solvent that possesses a heterogeneous composition of ions, chaperones, salts and low molecular weight compounds [168]. As the solvent environment determines the folding state of a protein, studies have revealed its manipulation as a strategy for avoiding diseases that result from defects in protein folding [169,170,171]. Cells often face environmental insults that are both extrinsic (pH, high salts, extremes of temperature, etc.) and intrinsic (high concentration of denaturants such as urea), which emerge as challenges to folding proteins into functionally active conformational states [168,172,173]. Failure to cope with cellular challenges and hostile environments often leads to proteopathies (protein destabilization followed by aggregate or amyloid formation) [174,175]. Taking AD and PD into consideration, protein intermediates that arise due to mutations are kinetically unstable, and often lead to aggregate or amyloid formation(a state that can never be reversed to native conformation) [171,176].

As a quality control system, cells or organisms overcome such hostile environments by enhancing the accumulation of small organic (amino acids and their derivatives, methylamines, etc.) entities, referred to as osmolytes. Increasing the values of the *T*m (melting temperature) and *C*m (melting concentration) of proteins, the correction of folding defects via reduction in the aggregation of proteins is the major attribute of osmolytes [177,178,179,180,181,182,183,184,185,186]. The osmoregulatory property of taurine corresponds to its presence at higher concentrations in cells exposed to higher levels of oxidants [187,188,189]. The anomalous behavior of taurine (increasing under oxidative stress and decreasing under hypo-osmotic conditions) forms part of the mechanism by which it imparts protection to cells from extra-stretching under an osmotic imbalance condition [190]. Capable of establishing water-mediated interactions, taurine gives stability to proteins against stress conditions capable of causing protein denaturation [191,192,193,194,195]. NMR (Nuclear magnetic resonance spectroscopy) studies revealed the involvement of taurine in the refolding of denatured proteins [196], while spectroscopic and calorimetric studies revealed its role in increasing the thermal stability of lysozyme [197,198]. Khan et al. revealed the role of taurine in counteracting the denaturing of proteins by urea, via increasing the stability of the protein for the maintenance of its function [199]. In their studies of measuring the enzymatic activity and thermal stability of proteins, they found the effect of taurine to be protein-specific. In addition to the protein stabilizing effect, taurine, at millimolar concentrations, is reported to play a role in preventing aggregation of the proteins [7,200,201].

Progression of proteins from the monomeric to fibrillar stage accompanies their aggregation. Representing a significant hallmark of NDs, this serves dual purpose, in the early-stage diagnosis of disease as well as in developing therapeutics for them. In the case of AD, taurine supplementation exerts its therapeutic effect by reducing Aβ aggregation [202]. Kim et al. demonstrated that oral administration of taurine to a APP/PS1 transgenic mouse model relieves its cognitive defects via decreasing the Aβ levels [203]. A similar effect of relieving cognitive effects was observed in the studies on the AD mouse model [202]. Attenuation in the aggregation of α-synuclein was observed in the PD mouse model developed by intoxication of paraquat and maneb [154]. Thioflavin T(ThT) emission monitoring of glucagon fibrillation via enhancement in osmolytes revealed taurine-mediated protection, observed as extension in the lag phase [204].

### 6.3. As Inhibitory Neuromodulator

The release of taurine as part of neurotransmission seems independent of the Ca^2+^ influx, as no vesicular transporter has been reported for taurine [205]. Considering the importance of taurine in the CNS, its release occurs via volume-sensitive organic anion channels, or through a mechanism that involves reversal of Tau-T functioning [206]. Taurine-mediated modulation of voltage gated Ca^2+^ channel functioning involves the binding of taurine to GABA/glycinergic receptors, which results in neuronal hyperpolarization. As basal unstimulated taurine release is low in the neonatal stage, various stimuli (such as hypoosmotic stimulation, volume change, glutamate, adenosine, etc.) trigger its release from the immature neural cortex [207,208,209]. As a potent neuroprotectant, taurine buffers the toxic effect in the CNS that arises as a result of an imbalance between inhibitory (e.g., GABA) and excitatory (e.g., glutamate) neurotransmitters [210]. On the one hand, while it protects the CNS from excitotoxicity by glutamate, it on the other hand prevents neuronal hypertoxicity by reducing GABA levels or the activity of the GABA receptors. Taurine acts as a weak agonist of GABA (GABA_A_, ionotropic GABA_B_ and metabotropic GABA_C_) receptors; it can therefore replace GABA for binding to the receptor and inhibiting neuronal excitability [15,210,211]. Regulation of the GABA_A_ receptor is complex. Acute taurine administration has an activator effect on the GABA_A_ receptor and the chronic taurine level that leads to downregulation of the GABA_A_ receptor, causing upregulation of the glutamate decarboxylase that catalyzes the rate limiting step reaction in GABA biosynthesis. Additionally, taurine acts as a partial agonist of glycine and NMDA (ionotropic glutamate receptor subtype) receptors [211,212]. Together, the operation of the complex interactive network, between taurine and the GABAergic and Glycine and/or NMDA receptors, largely defines its functionality in the CNS. 

### 6.4. Energy Metabolism Modulator

Taurine plays a vital role in energy metabolism; it acts as a key regulator, to maintain the production level of superoxides and oxidative phosphorylation. The ratio of NADH/NAD^+^ is raised by its deficiency, which effects the activity of complex I, resulting in a disturbance of the energy metabolism and oxidative stress via respiratory chain impairment, and also leads to the inactivation of 3-NADH-sensitive enzymes (α-ketoglutarate dehydrogenase, isocitrate dehydrogenase and citrate synthase) [213]. The pyruvate oxidation decreases due to taurine deficiency, and the activity of pyruvate dehydrogenase is stopped due to the elevating ratio of NADH/NAD^+^, which results in pyruvate deficiency due to the substantial conversion of pyruvate to lactate. In taurine-deficient hearts, the oxidation of glucose is declined, which in turn affects the biosynthesis of ATP. In the human liver, taurine biosynthesis is very low, and the diet is its main source. As per the study of Jeejeebhoy et al. (2002), patients with heart failure have been found taurine-deficient, and so for cardiovascular diseases it is considered as a therapeutic agent by providing its supplements to patients for the restoration of taurine levels, which results in proper contractile functions [214]. 

### 6.5. As ER Stress Modulator

ER stress, having its background in the misfolding of proteins, oxidative stress and the enhancement of intracellular Ca^2+^, interferes with signaling across neurons, which ultimately progresses to neuronal cell death. Stress-mediated activation of unfolded protein response (UPR^ER^) relieves cells of the stress condition through the activation of downstream signaling across three cascades—PERK, IRE1 and ATF6—towards restoration of the balance between synthesis/folding and the degradation of proteins. The activation of signaling cascades is mediated by dissociation of glucose regulated protein-78 (GRP-78) from PERK, IRE1 and ATF6,which initiates downward signaling in order to overcome ER stress. Under prolonged stress conditions, UPR fails in restoring the correct folding of proteins, and as such directs cells to apoptosis via the activation of pro-death components, such as the C/EBP homologous protein (CHOP), Caspase 12 and JNK [215,216,217]. Taurine is believed to be involved in restoring current folding of proteins, either through reduction in oxidative stress or through providing suitable osmotic conditions for proteins to fold [218]. As a neuro-protectant, taurine restores the structural integrity and functionality of ER through the reduction of intracellular Ca^2+^ levels and Ca^2+^-mediated oxidative stress, as well as the Bax/Bcl-2 ratio [150,219]. 

### 6.6. As Neuroinflamatory and Synaptic Loss Modulator

Taurine supplementation has been found to reduce the secretion of TNFα, IL-1α, IL-1β, IL-6, etc [220]. The effect was observed as a decrease in the expression of inflammatory stress markers. As neuroinflammation and synaptic loss pertains to activation of glial cells and the release of proinflammatory cytokines, inactivation of the microglia-mediated inflammation and activation of the NOX2-NF-kB pathway count as taurine-mediated neuroprotection effects [154]. For initiation of the neuroinflammatory cascade, intracerebral hemorrhage(ICH) plays a significant role, however, administration of a high dose of taurine in ICH model rat ameliorates white matter injury and neuronal damage. The effect was associated with the reduction of inflammatory mediators expression, glial activation, neutrophil infiltration and enhanced expression of CBS (cystathionine-β-synthase), etc [12]. In the maneb- and paraquat-induced mice model of PD, taurine inactivated microglia-mediated neuroinflammation, marked by downregulation of proinflammatory cytokines such as TNFα, IL1β, etc [154]. In AD, Aβ-induced inflammation is limited by reactive astrocytes. In the wild type and transgenic mice models of AD, oral administration of taurine induced increases in a number of reactive astrocytes [203]. The mtSOD1(G93A) transgenic cell line model of ALS also responds to taurine, with regards to neurotoxic injury [155]. 

### 6.7. As Ca^2+^ Homeostasis and Apoptotic Modulator

Glutamate, an excitatory neurotransmitter in the CNS, plays an important role in the survival and differentiation of neurons, besides maintaining neuronal plasticity for smooth synaptic transmission [221,222,223]. It was found that excessive amounts of extracellular glutamate induce cellular damage, which progresses to cell death via increases in the amount of intracellular free Ca^2+^ [224]. Taurine exerts its neuroprotective effect through maintenance of the structural integrity of the membrane that leads to decreases in the intracellular Ca^2+^ levels [225]. It prevents entry of Ca^2+^ into neurons via interference with the L, N and P/Q type of Ca^2+^ channels [226], besides formulating the operation of a reverse module Na^+^/Ca^2+^ exchanger [224,225,227]. As its indirect mode of operation, taurine enhances the activity of sarcoplasmic Ca^2+^-ATPase associated with the maintenance of cytosolic Ca^2+^ homeostasis, via uptake of cytosolic Ca^2+^ [228].

Glutamate-mediated accumulation of intracellular Ca^2+^ in mitochondria leads to increases in the production of ROS [229]. Together, ROS-mediated oxidative stress and enhanced Ca^2+^ triggers breach the mitochondrial membrane’s permeability, there by leading to the release of pro-apoptotic factors that ultimately causes cell progression to apoptosis [230]. In this process, cell progression to the apoptotic process is regulated by the balance of bcl-2-like protein 4 (Bax) to B-cell lymphoma 2 (Bcl-2) proteins. An enhancement of the intracellular Ca^2+^, mediated by glutamate, activates calpain (Ca^2+^-dependent protease), which is capable of cleaving Bcl-2. Simultaneously, glutamate induces dimerization of Bax via conformational changes in the structure of Bax, which causes the release of cytochrome C from mitochondria. With non-functional Bcl-2 (inactivated by calpain cleavage), Cyt C-mediated activation of Apaf-1 causes downstream signaling along the caspase cascade, thereby promoting apoptosis [231]. Together, glutamate-mediated increases in Bax and Bcl-2 promote apoptosis, while a decline of Bax to Bcl-2 ratio mediated by taurine prevents the progression of cells to apoptosis [231,232]. 

## 7. Conclusions

The aggregation of misfolded proteins that leads to the generation of plaques, tangles, Lewy bodies, etc., and their deposition in different cell subsets of the brain and in the extracellular milieu, finally proceeds to the development of different NDs. Despite the fact that studies are performed on different fronts to understand disease occurrence, and as such disease progression that affects normal brain function, there still lies a void in understanding the contribution of risk factors, the genetic aspects of occurrence of the diseases, and the development of potent therapeutics that could combat these devastating brain diseases. Mitochondrial dysfunction, ER stress, neuroinflammation and synaptic loss with subsequent neuronal death are considered as the foremost causes in the development of NDs. In the search for effective therapeutic possibilities, taurine—an osmolyte with wide occurrence in humans—has proven its ability to promote protein folding under stress conditions. It effectively mitigates the severity of consequences that arise due to protein misfolding, and thereby keeps a check on the progression of brain diseases such as AD. The remarkable properties of taurine as an antioxidant molecule, as a stabilizer in regulating protein folding/unfolding, as a modulator of apoptosis and in Ca^2+^ homeostasis, helps in attenuating the symptomology of misfolded protein aggregation. As a neuroprotective molecule, its alleviation of protein aggregation leads to improvement in neuronal function, thereby averting the neuronal damage that reduces brain functioning in different NDs. Further studies are needed to gain a deeper insight into taurine functioning, and to investigate its mode of operating and mechanism of protection in combating the occurrence, and as such progression, of different NDs. This would pave the way for researchers working in the field to developing potent therapeutics for employment in overcoming the plethora of different NDs. 

## Figures and Tables

**Table 1:** Role of Taurine in Neurodegeneration.

Disease	Hallmark of Disease	Taurine Effect	Reference
**Alzheimer’s disease**	Cerebral plaques consisting of *β*-amyloid peptides (Aβs) and intracellular neurofibrillary tangles (NFTs), mainly composed of hyperphosphorylated tau	Induces the synaptic potentiation, antioxidant property, inhibits neuronal death by increasing inhibitory neuro transmission via GABA_A_ and glycine receptor stimulation, suppresses mPTP opening and reverse mitochondrial function, attenuates A*β*-induced Ca^2+^ and ROS generation, pores open, reduces the mitochondrial membrane potential and increases ATP production, prevents mitochondrial dysfunction, shifts the ratio of Bcl-2:Bax in favor of cell survival, inhibits the formation of the Apaf-1/caspase-9 complex (apoptosome), suppresses upregulation of Caspase-12 and CHOP, suppresses ATF6 and IRE1 pathway, acts as GABA and the GABA_A_ receptor agonists, inhibits the Na^+^/Ca^2+^ exchanger reverse mode, inhibits L-, P/Q-, N-type voltage-gated calcium channels, prevents Ca^2+^ influx through NMDA receptor calcium channels, inhibits calcium release	[148,149,150,151,152]
**Parkinson’s disease**	Loss of dopaminergic nigrostriatal neurons, intra-cytoplasmic Lewy bodies (LBs), intra-axonal Lewy neurites (LNs)	Scavenges ROS by inducing the activity of endogenous anti-oxidants, catalases and glutathione peroxidase (GSHPx), reduces mitochondrial ROS to promote normal functioning by increase in anti-oxidant protection, suppresses upregulation of Caspase-12 and CHOP, suppresses ATF6 and IRE1 pathway, suppresses microglial M1 polarization via NOX2-NF-κB pathway	[150,153,154]
**Amyotrophic lateral sclerosis**	Neuronal death (motor) in the nervous system, mutations in the protein SOD1	Neuroprotective effects, against excitotoxicity induced by glutamate in motor neuronal cell lines, protects motor neuron from oxidative stress	[155,156]
